# Oviductal extracellular vesicles from women with endometriosis impair embryo development

**DOI:** 10.3389/fendo.2023.1171778

**Published:** 2023-06-20

**Authors:** Yuehan Li, Lei Cai, Na Guo, Chang Liu, Meng Wang, Lixia Zhu, Fei Li, Lei Jin, Cong Sui

**Affiliations:** ^1^ Reproductive Medicine Center, Tongji Hospital, Tongji Medical College, Huazhong University of Science and Technology, Wuhan, China; ^2^ Nanjing Drum Tower Hospital, The Affiliated Hospital of Nanjing University Medical School, Nanjing, China

**Keywords:** endometriosis, extracellular vesicles (EVs), fallopian tube, embryo development, oxidative phosphorylation

## Abstract

**Objective:**

To investigate the influence of oviductal extracellular vesicles from patients with endometriosis on early embryo development.

**Design:**

*In vitro* experimental study

**Setting:**

University-affiliated hospital.

**Patients:**

Women with and without endometriosis who underwent hysterectomy (n = 27 in total).

**Interventions:**

None.

**Main outcome measures:**

Oviductal extracellular vesicles from patients with endometriosis (oEV-EMT) or without endometriosis (oEV-ctrl) were isolated and co-cultured with two-cell murine embryos for 75 hours. Blastocyst rates were recorded. RNA sequencing was used to identify the differentially expressed genes in blastocysts cultured either with oEV-EMT or with oEV-ctrl. Kyoto Encyclopedia of Genes and Genomes (KEGG) pathway enrichment analysis were performed to identify potential biological processes in embryos that oEV-EMT affects. The functions of oEV on early embryo development were determined by reactive oxygen species (ROS) levels, mitochondrial membrane potentials (MMP), total cell numbers, and apoptotic cell proportions.

**Results:**

Extracellular vesicles were successfully isolated from human Fallopian tubal fluid, and their characterizations were described. The blastocyst rates were significantly decreased in the oEV-EMT group. RNA sequencing revealed that oxidative phosphorylation was down-regulated in blastocysts cultured with oEV-EMT. Analysis of oxidative stress and apoptosis at the blastocysts stage showed that embryos cultured with oEV-EMT had increased ROS levels, decreased MMP, and increased apoptotic index. Total cell numbers were not influenced.

**Conclusion:**

Oviductal extracellular vesicles from patients with endometriosis negatively influence early embryo development by down-regulating oxidative phosphorylation.

## 1 Introduction

Endometriosis is a complex clinical syndrome with a prevalence of 5% to 15% in women of reproductive age, characterized by the presence of endometrial tissue outside the uterus (1). The symptoms include chronic pelvic pain, dyspareunia, and dysmenorrhea. Infertility occurs most frequently among all clinical manifestations of endometriosis, and approximately 50% of patients present with decreased fertility (2).

The reason why endometriosis impairs women’s fertility hasn’t been fully characterized. Several mechanisms have been raised to explain the phenomenon, including an inflammatory pelvic environment, ovulatory abnormalities, and altered functions of the endometrium (3). Inflammatory microenvironment was found to negatively influence gamete and embryo in endometriosis, as it may impair sperm function, decrease oocyte fertilization rate and harm early embryo development (4–6). Toxic factors are identified in peritoneal fluid and may enter the lumen of Fallopian tubes (7). In women who undergo assisted reproductive technology (ART), there is no difference in pregnancy outcomes in patients with endometriosis compared with patients undergoing ART for male factor infertility and non-infertile patients after transferring euploid embryos (8), and endometriosis does not affect blastocyst rates (9) or the live birth rates (10). These results indicate that ART might be the hope for patients with endometriosis-associated infertility who wish to conceive. However, how endometriosis affects fertility in natural conception is still poorly understood.

After fertilization, embryos undergo a series of cleavages within the maternal oviduct (or Fallopian tube in humans). Ciliated cells and secretory cells from the epithelial line in the oviduct, together with the oviduct fluid secreted by these cells, constitute the environment for early embryo development (11–14).

Extracellular vesicles (EVs) have been recognized to mediate intercellular interactions by transferring cargo, including RNAs and proteins (15), and they are transferrable even between species (16). EVs have been isolated from various biofluids, including follicular, oviduct, and uterine fluid (17). EVs from the reproductive system have been reported to influence embryo viability and development capacity *in vitro (* 18). For instance, researchers showed that EVs secreted by endometrial cells of patients suffering from recurrent implantation failure is detrimental to the embryos (19). Also, it has been reported that oviductal EVs play an essential role in oviduct-embryo interactions (18, 20, 21).

In the current study, we hypothesize that the EVs within the Fallopian tube in patients with endometriosis (oEV-EMT) might contribute to the impaired development of embryos. Subsequently, we tried to discover the influence EVs may exert on embryo development respecting blastocyst rate and the transcriptome of the embryos.

## 2 Methods

### 2.1 Ethics approval, participants, and sample acquisition

The current study was approved by the Ethics Committee of Tongji Hospital (No. TJ-IRB20210838). The EMT patients were indicated for the surgery because of severe pelvic pain or adenomyosis by ultrasound, and were all diagnosed with peritoneal endometriosis by laparoscopic inspection. Pelvic superficial endometriosis and deep endometriosis were both included. The control patients had at least given one full-term live birth and had no history of endometriosis, and they underwent laparoscopic hysterectomy because of cervical intraepithelial neoplasia (CIN), and no pelvic pathophysiologic conditions were reported before or after the surgery inspection. The exclusion criteria for all groups were as follows: received hormone treatment three months before the surgery; pregnancy; any indication for malignant diseases (including previous history or other tests showing signs). Cycle phases were recorded according to the patient’s reports. Between August 2021 and February 2022, 27 women aged between 34 to 43 were recruited for the study. The samples were collected in the operation room, immediately after the Fallopian tube and the uterus were resected. We used a 50 mL syringe that contained sterile PBS (Servicebio, Wuhan, China) to flush the lumen of the Fallopian tubes. Then we collected the flushing fluid (Fallopian tube fluid) into sterile centrifuge tubes for further analysis as reported (22). The Fallopian tube tissue was collected at the same time, and was conserved in cold PBS. The Fallopian tube fluid and tissue samples were transferred to the laboratory immediately and stored for later processing.

### 2.2 Isolation of oviductal extracellular vesicles

Extracellular vesicles (EVs) were isolated by the ultracentrifugation method, as described before (21). In brief, the Fallopian tubal fluid was centrifuged at 1,500 × g for 15 minutes twice, and the supernatant was centrifuged at 16,000 × g for 30 minutes at 4 °C. Then the sediment was discarded, and the supernatant was filtered through a 0.22-μm filter. Afterward, the fluid was ultracentrifuged at 120,000 × g for 90 minutes at 4 °C. The pellet was washed with clean PBS and then ultracentrifuged at 120,000 × g for 90 minutes at 4 °C. The pellets were resuspended in 30 μL PBS and stored at -80 °C.

### 2.3 Western blotting

EVs or tissue samples were lysed with radioimmunoprecipitation assay (RIPA) and proteinase inhibitor cocktail (Servicebio, Wuhan, China) at 4°C for 30 minutes. Then the lysates were centrifuged at 12,000 g for 20 minutes at 4°C. The protein concentration of EVs and Fallopian tube tissue samples were measured using a BCA Protein Assay Kit (Servicebio, Wuhan, China). 5μg of each sample was loaded for electrophoresis, using SDS/PAGE (10% gel), followed by transferring to PVDF membrane (Merck Millipore, Burlington, MA, USA). Then, the membrane was blocked using 5% skimmed milk for 1 h at room temperature, followed by washing with TBS for 15 min. Afterward, the membrane was incubated with the primary antibodies at 4 °C overnight. The primary antibodies used for immunostaining were TSG101 (1:1000; Abclonal, Woburn, MA, USA) and CD9 (1:1000; Abcam, Cambridge, UK), and the secondary antibodies were goat anti-rabbit labeled by horseradish peroxidase (1:2000; Servicebio, Wuhan, China). The membrane was incubated with the secondary antibodies for 1h at 37 °C and then was immersed in an electrochemiluminescence (ECL; Absin, Shanghai, China). The signals were detected using a Gene Gnome XRQ chemiluminescence imaging system (Syngene, Bengaluru, India).

### 2.4 Transmission electron microscopy

Each EV sample was deposited on a precoated carbon electron microscopy grid for TEM. Then the grids were labeled with 2% uranyl acetate (in double-distilled water). Grids were examined and pictured using a transmission electron microscope (Carl Zeiss, Oberkochen, Germany).

### 2.5 Nanoparticle tracking analysis

Samples were analyzed using an NTA instrument, zetaview (Particle Metrix, Inning am Ammersee, Germany). EV samples were diluted with PBS at a ratio of 1:1000 to reach the concentration recommended for the measurement (3 × 10^8^ to 1 × 10^9^ particles/mL). For each sample, three videos of 60 seconds were recorded and analyzed, and the particle concentration and sizes were recorded.

### 2.6 Animals and treatment

All experiments followed the Tongji Hospital Guide for the Care and Use of Laboratory Animals (approval number: TJ-202111004). Sixty female (6-8 weeks of age) and ten male (8-10 weeks of age) mice were used in the study. Female mice were intraperitoneally injected with 10 IU of pregnant mare serum gonadotropin (PMSG; Solarbio, Beijing, China), followed by 10 IU of human chorionic gonadotropin (hCG; Livzon, Zhuhai, China) 48 hours later. After the injection of hCG, two female mice were mated with one male mouse for one night. Thirty-six hours post the hCG injection, the zygotes were isolated from oviducts.

Chatot-Ziomek-Bavister (CZB; AIBI bio, Nanjing, China) medium was used as basic embryo culture medium in the current study. oEV-EMT or oEV-ctrl were added to CZB medium to obtain the culture media for the treated groups. As oEVs were suspended in PBS after the ultracentrifugation, a small volume of this primed PBS was mixed with CZB medium to obtain the final concentration of 1×10^10^ particles/mL. The culture media of the two groups(CZB with oEV-EMT, CZB with oEV-ctrl) were divided into 20-μL droplets covered with mineral oil, and each droplet contained ten zygotes, and the embryos were subjected to further study. The embryo culture media were prepared and equilibrated for 2 hours before the embryos were loaded. And the embryos were cultured at 37°C under an atmosphere of 5% CO_2_, 5% O_2_, and 90% N_2_ for 75 hours. For all following experiments, at least 30 embryos per group were used in each experiment, and the experiments were repeated at least three times.

### 2.7 Endocytosis of extracellular vesicles by embryos

To investigate whether human Fallopian tube-derived oEVs could be taken up by murine embryos, we used 3,3’-dioctadecyloxacarbocyanine perchlorate (DiO; Beyotime, Shanghai, China) to dye the membrane of oEVs (19). In brief, EMT-oEVs, ctrl-oEVs, or PBS (negative control) were incubated with DiO (10 μM) for 30 minutes. Then the dyed oEVs or negative control were resuspended in PBS and ultracentrifuged at 120,000 g for 80 minutes. Labeled oEVs or negative control were co-cultured with 2-cell embryos for 4 hours. oEVs uptake was observed under a fluorescence microscope (Carl Zeiss, Oberkochen, Germany).

### 2.8 RNA extraction, library construction, and RNA sequencing procedures

Total RNA was isolated from three replicates of 10 blastocysts using the RNeasy Micro Kit (Qiagen, Hilden, Germany) according to the manufacturer’s instructions. The quality and concentration of the RNA samples were determined by 2100 Bioanalyser (Agilent Technologies, Santa Clara, CA, USA). Transcriptome library preparation was done following the TruSeq™ RNA sample preparation Kit from Illumina (San Diego, CA, USA). In brief, messenger RNA was isolated using the polyA selection method by oligo (dT) beads, followed by fragmentation in the buffer. Afterward, double-stranded cDNA was synthesized by a SuperScript double-stranded cDNA synthesis kit (Invitrogen, Waltham, MA, USA) with random hexamer primers (Illumina, San Diego, CA, USA). Then the synthesized cDNA was subjected to end-repair, phosphorylation, and ‘A’ base addition according to Illumina’s library construction protocol. Libraries were selected for cDNA target fragments of 300 bp on 2% Low Range Ultra Agarose followed by PCR amplified using Phusion DNA polymerase (NEB, Ipswich, MA, USA) for 15 PCR cycles. After quantified by TBS380, the paired-end RNA-seq sequencing library was sequenced with the Illumina HiSeq X ten/NovaSeq 6000 sequencer.

The raw reads were aligned to genome sequences, and the mapped reads of each sample were assembled by StringTie (https://ccb.jhu.edu/software/stringtie/index.shtml?t=example) in a reference-based approach (23). To identify the differentially expressed genes (DEGs) between the groups of samples, the expression level of each transcript was calculated according to the transcripts per million reads (TPM) method. Moreover, differential expression analysis was performed using the DESeq2, and genes were considered significantly differentially expressed if the Benjamini-Hochberg adjusted *p*-value was ≤ 0.05 and the absolute value of the log_2_ foldchange was ≥ 1. In addition, functional-enrichment analysis, including GO and KEGG, was performed to identify which DEGs were significantly enriched at Bonferroni-corrected *p* value ≤ 0.05 compared with the whole-transcriptome background. GO functional enrichment and KEGG pathway analysis were carried out by Goatools (https://github.com/tanghaibao/Goatools) and KOBAS (http://kobas.cbi.pku.edu.cn/home.do) (24).

### 2.9 Real-time-PCR

Blastocysts were collected and immediately reverse-transcribed using Single-Cell Sequence-Specific Amplification Kit (Vazyme, Nanjing, China) according to the manufacturer’s protocol. Subsequent real-time RT-PCR was performed on a Roche Light Cycler 480 Instrument II PCR System with TB Green^®^ Premix Ex Taq™ (Tli RNaseH Plus) (TaKaRa, Kusatsu, Japan). The expression level of each gene was normalized by *Gapdh* and was calculated using the 2^−△△Cq^ method. Five blastocysts per group were used for each reaction, and each experiment was repeated at least three times. The primer sequence is provided in **Supplementary Table 1**.

### 2.10 ROS measurement

To measure ROS levels by fluorescence, the stock solution of 2’, 7’ - dichlorodihydro-fluorescein diacetate (DCHF-DA; Sigma, St. Louis, MO, USA) was used to dye the embryos. DCHF-DA was diluted to 10 μM with CZB medium, and embryos in each group (n = 10 - 15) were dyed at 37°C for 20 minutes. The embryos were washed three times in CZB and immediately observed under a fluorescence microscope (Carl Zeiss, Oberkochen, Germany). All images were analyzed using Image J software.

### 2.11 Measurement of MMP

To investigate the mitochondrial membrane potential (MMP), living blastocysts were incubated with CZB supplemented with 2 μg/L JC-1 Dye (Service bio, Wuhan, China) for 30 min at 37 °C according to the manufacturer’s instructions. After washing in CZB twice, embryos were observed under a fluorescence microscope (Carl Zeiss, Oberkochen, Germany). Each blastocyst was observed through the TRITC channel (red fluorescence) and the FITC channel (green fluorescence). All images were analyzed using Image J software, and the ratio of aggregated JC-1 (red fluorescence) to monomeric JC-1 (green fluorescence) was calculated to reflect changes in MMP.

### 2.12 TUNEL assay

After co-cultured with oEVs-EMT, oEVs-ctrl, or PBS for 75h, the blastocysts were fixed with 4% paraformaldehyde (Servicebio, Wuhan, China). To investigate the proportion of the apoptotic cells in blastocysts, the terminal deoxynucleotidyl transferase dUTP nick end labeling (TUNEL) assay was performed using the TMR (red) Cell Apoptosis Detection Kit (Servicebio, Wuhan, China) according to the manufacturer’s instructions. Then the embryos were dyed with diamidino-2-phenylindole (DAPI; Servicebio, Wuhan, China). Dyed blastocysts were mounted on a glass slide and observed under a fluorescence microscope (Carl Zeiss, Oberkochen, Germany). The cell numbers were counted, and the percentage of apoptosis cells was calculated as the TUNEL-positive cell number divided by the total cell number of the blastocyst.

### 2.13 Statistical analysis

Experiments were performed at least three times. Statistical tests were performed using Graph Pad Prism 8.0 (San Diego, CA, USA). Quantitative variables were shown as mean ± SEM. Blastocyst rates were analyzed using the chi-square test. To determine statistical differences in maternal age and body mass index (BMI), student t-tests were performed. For relative gene expression, relative expression level of ROS, MMP, total cell number, and apoptotic cell proportion, one-way analysis of variance (ANOVA) was performed. *p* < 0.05 was considered to be significantly different.

## 3 Results

### 3.1 Clinical characteristics and the characterization of oEVs from endometriosis patients and controls

**Figure 1:**
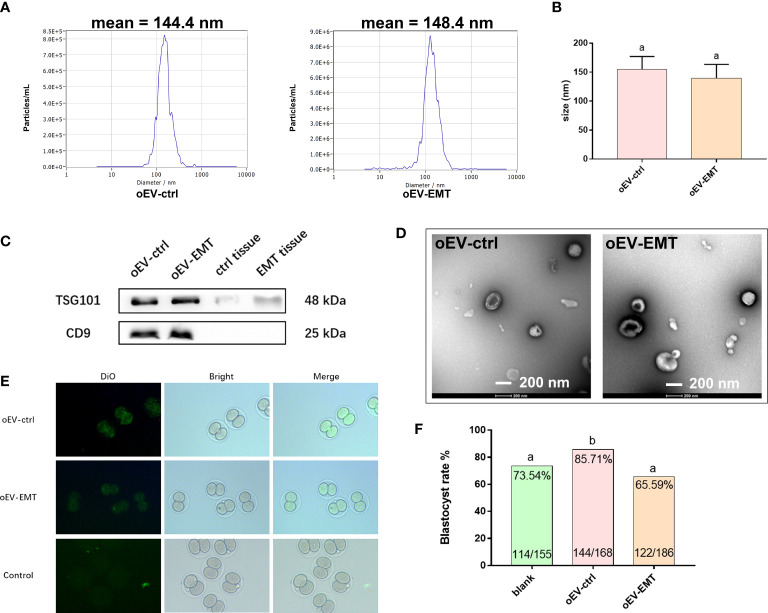
Characterization of oEV-EMT and oEV-ctrl. **(A)** The sizes of oEV-EMT and oEV-ctrl were evaluated using NTA. **(B)** The diameters of oEV-EMT and oEV-ctrl samples are similar. **(C)** The expression of EV markers was detected by Western blotting, and Fallopian tube tissue was used as a control. **(D)** Representative TEM images of oEV-EMT and oEV-ctrl are shown. The typical bilayer of the EV structure can be seen. **(E)** Pictures of DiO-labelled oEV-EMT, oEV-ctrl, or blank control being taken in by two-cell murine embryos. **(F)** Blastocyst rate in each group and the number of embryos were given. oEV, oviductal extracellular vesicles; EMT, endometriosis; ctrl, control; NTA, nanoparticle tracking analysis; TEM, transmission electronic microscopy, DiO, 3,3’-dioctadecyloxacarbocyanine perchlorates. Different superscripts per column **(A, B)** represent statistical differences (*p* < 0.05) between groups.

Demographics of the patients are characterized in **Supplementary Table 2**. The morphology and distribution of oEVs were detected using NTA and TEM. NTA profiles showed peaks at around 144.4 nm in the control group and 148.4 nm in the EMT group (**Figure 1A**). There’s no difference in the diameters between the groups (**Figure 1B**). Western blotting showed the presence of classic EV protein markers in samples from both groups, including CD9 and TGS 101. Fallopian tube epithelial cells from endometriosis patients and controls were used as control samples, and CD9 and TSG101 were almost undetectable in the control samples (**Figure 1C**). TEM images showed the typical bilayer structure of the EVs from both the groups at the expected size (**Figure 1D**).

### 3.2 oEV-EMT impairs the development of embryos

oEVs were confirmed to be taken up by murine embryos, as green fluorescence signals could only be seen in oEV-EMT- or oEV-ctrl-co-cultured embryos, not in the blank control group (**Figure 1E**). Blastocyst rate is lower in the oEV-EMT group comparing to the oEV-ctrl group (65.59% vs. 85.71%) (**Figure 1F**). Notably, oEV-ctrl significantly improves the blastocyst rate from 73.54% to 85.72%, compared to the blank control group. This result is consistent with our previous study, which showed that oEV-ctrl is beneficial for embryo quality *in vitro*.

### 3.3 oEV-EMT supplementation during *in vitro* culture altered embryonic transcriptome

**Figure 2:**
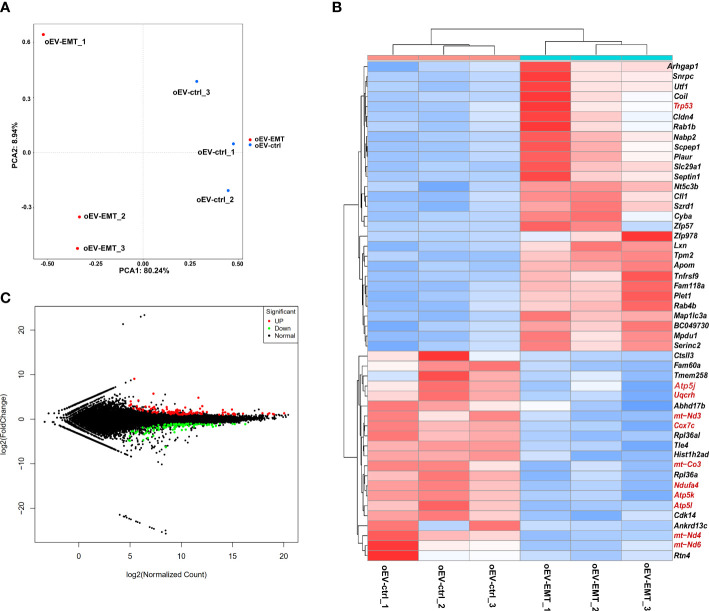
High-throughput sequencing showed differentially expressed genes between blastocysts treated with oEV-EMT or oEV-ctrl. **(A)** Principal component analysis. **(B)** MA plot shows all the genes in blastocysts from the two groups. Green and red dots denote down- and upregulated genes with FDR < 0.05. **(C)** Heatmap shows 29 most upregulated and 21 most downregulated genes. MA, M-versus-A plot. FDR, false discovery rate; oEV, oviductal extracellular vesicles; EMT, endometriosis; ctrl, control.

**Figure 3:**
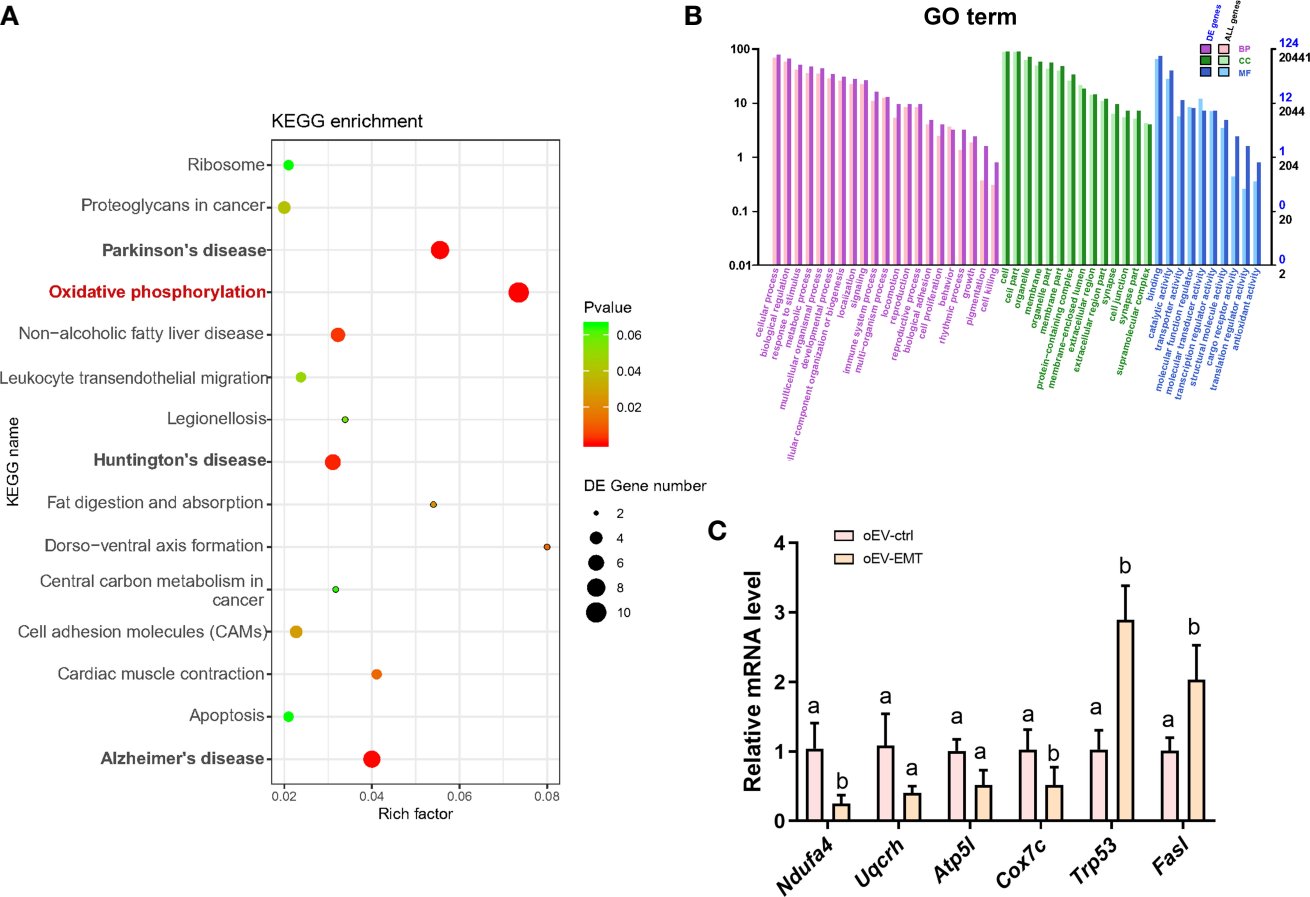
KEGG pathway enrichment and GO terms analysis of the differentially expressed genes. **(A)** The top 15 significant KEGG pathways are shown. Rich factor = (the number of DEGs in some KEGG pathway/the number of all DEGs that can be assigned to the KEGG database)/(the number of genes contained in a KEGG pathway/the total number of genes that can be assigned to the KEGG database). The greater the rich factor, the greater the degree of enrichment. **(B)** Bar plot showing GO terms with the number of differentially expressed genes for BP (biological process), CC (cell component), and MF (molecular function). **(C)** Validation of selected differentially expressed mRNAs by real-time RT-PCR. Different superscripts per column (a, b) represent statistical differences (*p* < 0.05) between groups. KEGG, Kyoto Encyclopedia of Genes and Genomes; GO, Gene Ontology; DEG, differentially expressed genes; oEV, oviductal extracellular vesicles; EMT, endometriosis; ctrl, control.

The principal component analysis showed blastocysts treated with oEV-EMT or oEV-ctrl display distinct linear distribution constructed by the first component (**Figure 2A**). Using DESeq2; we have found expression profiles of blastocysts from the two groups which are significantly different from one another. A total of 197 differentially expressed genes (DEGs) were identified; 111 were upregulated, and 85 were downregulated (**Figure 2B**, **Supplementary Table 3**). The heatmap showed the top 29 most upregulated and the top 21 most down-regulated genes (**Figure 2C**). To investigate the biological processes that contribute most to the differentially expressed gene profiles between the blastocysts treated with oEV-ctrl or oEV-EMT, we performed KEGG and GO enrichment on the 197 differentially expressed genes. KEGG analysis showed the most enriched pathway was oxidative phosphorylation (**Figure 3A**). Also, pathways of human neurodegenerative disorders, including Parkinson’s disease, Alzheimer’s disease, and Huntington’s disease, were highlighted. Enriched GO terms include metabolic process, developmental process, cell proliferation, transporter activity, cargo receptor activity, and antioxidant activity (**Figure 3B**). Using real-time RT-PCR, we confirmed that in the oEV-EMT-treated embryos, *Ndufa4* and *Cox7c* were significantly down-regulated, and *Trp53* and *Fasl* were significantly upregulated (**Figure 3C**). *Uqcrh* and *Atp5l* did not show a statistical difference.

### 3.4 Oxidative stress, mitochondrial membrane potential, and apoptotic analysis in oEV-treated blastocysts

**Figure 4:**
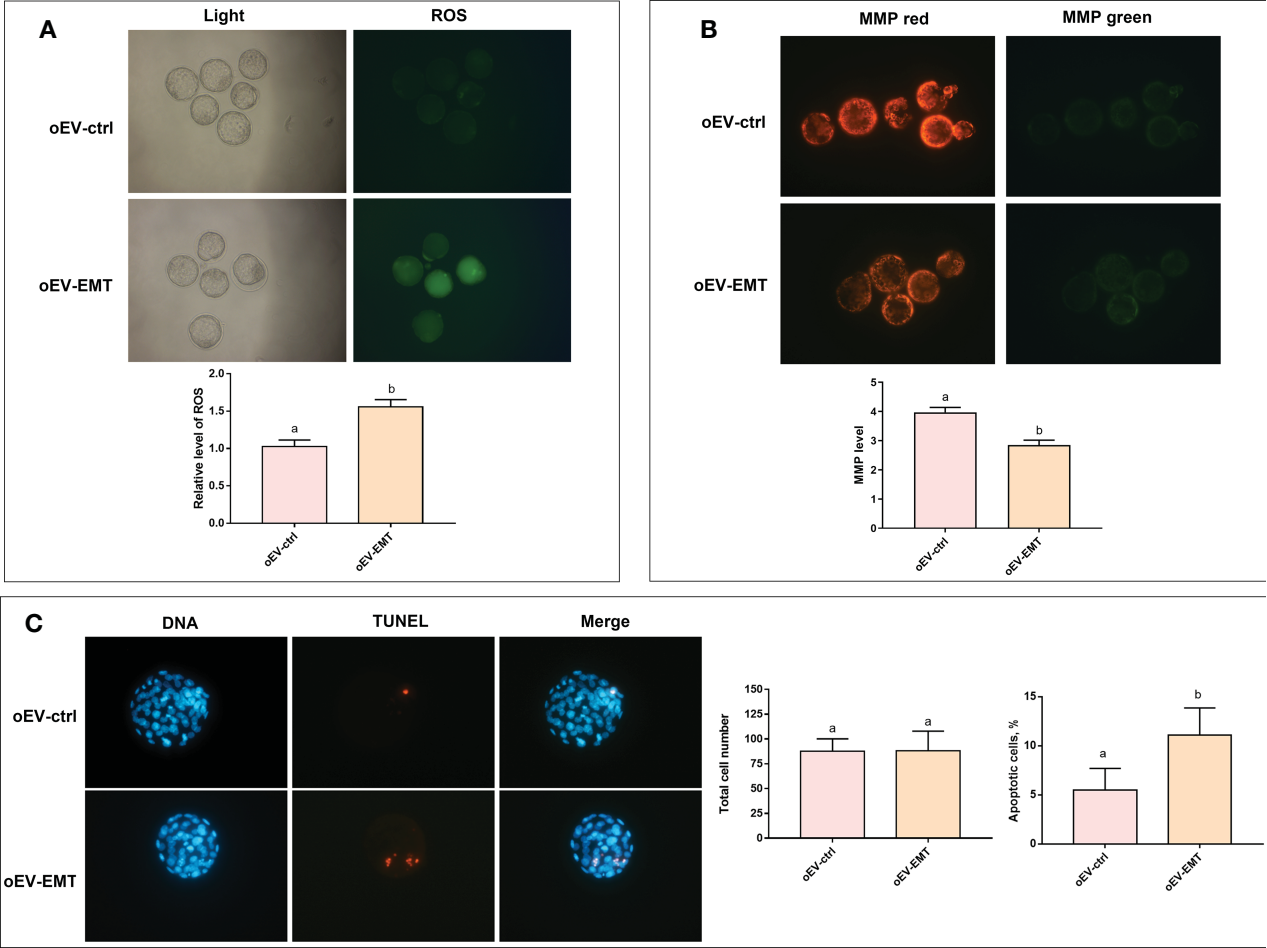
Effect of oEV-EMT on the relative level of ROS, MMP, and apoptotic cell numbers. **(A)** The fluorescence pictures and the relative levels of ROS (green fluorescence intensity value) were shown. **(B)** MMP, as measured by JC-1 staining, was shown as red/green fluorescence. **(C)** Representative images of apoptosis detected by TUNEL in blastocysts and the comparison of total cell number and apoptosis rates in blastocysts treated with oEV-EMT or oEV-ctrl are provided. Values indicated by different superscripts (a, b) are significantly different (*p* < 0.05). ROS, reactive oxygen species; MMP, mitochondrial membrane potential; TUNEL, terminal deoxynucleotidyl transferase-mediated dUTP nick-end labeling; oEV, oviductal extracellular vesicles; EMT, endometriosis; ctrl, control.

We checked the effects of oEV-EMT on oxidative stress and apoptosis in blastocysts. The ROS level in blastocysts treated with oEV-EMT was significantly higher than those treated with oEV-ctrl (1.556 ± 0.051 vs. 1.025 ± 0.056, *p*=0.039) (**Figure 4A**). MMP level was evaluated by staining blastocysts with JC-1. The MMP (referring to the ratio of red to green fluorescence) of oEV-EMT-treated blastocysts was significantly lower than oEV-ctrl treated blastocysts (2.846 ± 0.099 vs. 3.972 ± 0.096, *p*=0.028) (**Figure 4B**). Furthermore, as shown in **Figure 4C**, though the total cell number in blastocysts was not affected by oEV-EMT (87.769 ± 3.461 vs. 88.077 ± 5.523, *p*=0.963), oEV-EMT significantly increased the proportion of apoptotic cells in blastocysts (11.075 ± 1.391 vs. 5.475 ± 1.112, *p*= 0.003).

## 4 Discussion

This study provides evidence that EVs in the Fallopian tubes of women with endometriosis negatively affect embryonic development by down-regulating oxidative phosphorylation. Endometriosis is strongly associated with infertility. Several women with endometriosis would seek ART, including *in vitro* fertilization (IVF), for conception. Several studies have shown that endometriosis does not impair IVF outcomes in terms of live birth and clinical pregnancy, though women with endometriosis have lower oocyte yield in each cycle than women without endometriosis (9, 25). These results suggested embryo quality might be affected in women with endometriosis by the harmful environment in the Fallopian tube where fertilization and early embryo development happen. By circumventing the tubal microenvironment, the *in-vitro*-fertilized embryos can develop as well as in women without endometriosis (8). EVs have been recognized to contribute to disease pathophysiology in endometriosis (26–28), and whether EVs in endometriosis affect embryo development has not been studied. In this study, we constructed a co-cultured system using two-cell murine embryos and oEV-EMT or oEV-ctrl, as previously described (19). This co-culture system is reasonable because oviductal EVs are demonstrated to be conserved between humans and mice (22), and cargos in EVs are identified as transferrable between species, which means EVs produced by one species can be taken up by other species and function in the recipient cells subsequently (16).

First, we confirmed that murine embryos could take human-derived oEVs. We observed that oEV-EMT significantly decreased the blastocyst rate, compared to the oEV-ctrl group, though the characterizations of oEVs are not different between the groups. Significantly, the blastocyst rate in the oEV-ctrl group was significantly higher than in the control group. This result is consistent with our previous study, which suggested that oEVs from women without endometriosis or other pelvic inflammation improve embryo quality *in vitro* (29). Previous literature demonstrated that *in vitro* fertilized embryos show poorer developmental competence than their *in vivo* counterparts (30, 31). Also, as conventional *in vitro* culture media might epigenetically affect embryos (32), the addition of healthy oEVs that contain RNAs and proteins associated with chromatin modification would partly compensate for the alterations (33). On the other hand, oEVs from pathological conditions might negatively affect embryos. As EVs were not recognized as one of the main elements of oviductal fluid until recently, their roles as regulators of oviduct-embryo communications are mostly mysterious (34). In this study, the difference in blastocyst rate between the oEV-EMT group and the oEV-ctrl group showed oEVs in patients with endometriosis impaired embryo development compared with women without this condition. However, the blastocyst rates in the oEV-EMT group and the blank control group were not statistically different.

Further, high-throughput sequencing on blastocysts and subsequent KEGG analysis revealed decreased expression levels of genes related to oxidative phosphorylation in blastocysts cultured with oEV-EMT. Moreover, analysis of oxidative stress and apoptosis at the blastocysts stage showed that embryos cultured with oEV-EMT had decreased JC-1 ratio, increased ROS level, and increased apoptotic index.

During development, embryonic metabolism shifts from predominantly glycolytic to predominantly oxidative phosphorylation (35), and enhanced oxidative phosphorylation is critical for supporting high energy needs in preimplantation embryos. Our KEGG analysis showed that ‘oxidative phosphorylation’ is disturbed in oEV-EMT-treated blastocysts. Oxidative phosphorylation is one of the most indispensable metabolic pathways in which cells produce the majority of the adenosine triphosphate (ATP), and take place inside mitochondria. Pathways of neurodegenerative disorders, including Parkinson’s disease, Alzheimer’s disease, and Huntington’s disease, were also enriched, due to DEGs in these pathways are associated with oxidative phosphorylation and apoptosis, which are central aspects of these neurodegenerative diseases (36). We validated that some genes responsible for the electron transport chain were significantly downregulated in oEV-EMT-treated embryos, including *Ndufa4* and *Cox7c*, at the blastocyst stage. *Ndufa4* encodes a subunit of complex IV of the electron transport chain and was reported to promote oxidative metabolism and MMP and could inhibit ROS levels and promote tumor cells (37, 38). *Uqcrh* encodes a subunit of the mitochondrial complex III and is responsible for the electron transfer between cytochrome *C* and cytochrome *C*1 during oxidative phosphorylation (39). *Atp5l*, *Atp5j*, and *Atp5k* encode the components of mitochondrial ATP synthase. ATP synthase affects embryo development using catalyzing ATP synthesis through an electrochemical gradient of protons across the mitochondrial inner membrane during oxidative phosphorylation (35). *Cox7c* encodes a long-lived mitochondrial protein that forms a stable contact between complex I complex II and is required complex IV (40). Altogether, oEV-EMT-treated embryos showed a down-regulation of a gene series that encodes the proteins involved in mitochondrial transmembrane transport, which may debilitate general mitochondrial function, resulting in attenuated embryo development. Supplementing the transcriptomic analysis, we observed functional abnormalities on oEV-EMT-treated blastocysts, including decreased MMP and higher ROS levels. Several studies observed a similar phenomenon in murine embryos when co-cultured with peritoneal fluid from patients with endometriosis (6, 41) and suggested an impairment in embryo viability. Also, some studies found embryos would show a higher ROS level and decreased MMP after being cultured in less optimal conditions, like higher oxygen concentrations (42), or with lipotoxic gradients in culture media which simulated maternal metabolic disorders (43). The latter study proposed that mitochondrial-targeted antioxidants might help rescue development competence in embryos.

‘Apoptosis’ is another significantly enriched pathway closely relevant to embryo development. We validated that the expression level of *Trp53* and *Fasl* was upregulated in oEV-EMT-treated blastocysts, and the TUNEL assay showed an increased apoptotic index in oEV-EMT-treated blastocysts. Embryonic quality is generally associated with lower apoptotic rates, though apoptosis plays an essential role during early mammalian embryo development under physiological conditions (44, 45). Previous literature reported that animal-derived oEV from the normal condition would be beneficial for improving the quality of the *in-vitro*-fertilized embryos, appearing as increased total cell number and lower ROS level, and fewer apoptotic cells (45, 46). Our results suggested that oEV in pathological conditions, specifically endometriosis, may play a harmful role through similar mechanisms in an opposite way. Intriguingly, considering mitochondria-ROS crosstalk is pivotal for apoptosis induction (47), and apoptosis would result in MMP dissipation and excessive ROS formation, it is not clear whether the increased apoptosis in the oEV-EMT-treated embryos was caused by the excessive ROS or represented a direct effect of oEV-EMT, which requires further investigation.

We have shown no difference in oEV characterizations between the two groups, including vesicle size and ultrastructure. However, they cause different results in embryos. Several studies deciphered oEV cargo in animals, proved they carry various bioactive molecules, including proteins, lipids, microRNAs, and DNAs, and proved they hold the possibility of affecting the gene expression and behavior of gamete and embryos (18, 33, 48). Studies on endometriosis have recognized EV contents from patients’ plasma and peritoneal fluid (26, 27) and showed that the protein and RNA contents are associated with numerous biological processes and might contribute to disease pathophysiology. In our case, it is difficult to determine the critical factor or factors that are accountable for the results because any differentially expressed proteins, lipids, or nucleic acids might be the answer, and it requested numerous amounts of efforts involving high-throughput sequencing workload in the future.

The disease process of endometriosis can impair the oocyte quantity and quality for inflammatory conditions in the peritoneum. Proinflammatory factors that harm gametes, including cytokines and interleukin, have been identified in the peritoneal and Fallopian tubal fluid (49, 50). We showed for the first time that EVs also constitute bioactive factors in the tubal fluid which might negatively affect embryo development in endometriosis.

A particular strength of the current study is that the oEVs are directly isolated from human Fallopian tubal fluid instead of cultured cells. One possible limitation is that we were not able to conduct high-throughput sequencing in the blastocysts which had been cultured without oEVs. Blastocysts might show altered gene expression even in the oEV-ctrl group compared to the embryos cultured with solely culture media. Additionally, due to methodological and ethical limitations, we could not utilize human embryos as the study object, and oEVs may have different effects on human embryos. For a similar reason, the control group was CIN III patients, rather than healthy women. CIN is a precancerous disease, in which affected cells are considered restricted to the uterine cervix. Previous studies indicated that CIN history or treatments for CIN do not affect fecundability or obstetric outcomes (51, 52). Therefore, given ethical and logistical constraints, we believe that they provide the best available controls, although we cannot completely exclude that oEVs from CIN patients may show some differences with disease-free women. Notably, the clinical characteristics in this study showed no difference in gravidity and parity between EMT patients and controls, and this result seems to contradict the consensus that EMT patients have decreased fertility. We surmised that the small patient cohort in this study could explain this. Furthermore, cargos in oEVs may be under hormonal regulation, which is observed both in cows and pigs (33, 53). Though the fluctuation patterns and the content compositions are different among species (54), the situation might be much more complicated in humans. These conditions should be noticed when a more general conclusion is about to be drawn.

## 5 Conclusion

The present study compared the characterizations of the oEVs from women with or without endometriosis and showed that oEV-EMT negatively influences early embryo development. Altered transcriptome and decreased MMP, increased ROS levels, and apoptotic cell numbers were observed in blastocysts. Our results call for further effort in deciphering the protein and nucleic acid content in oEV-EMT and the mechanisms they affect embryos and gamete development *in vivo*, which would be meaningful for unrevealing the mystery of how endometriosis impairs human fertility.

## Data availability statement

The transcriptomics datasets generated during the current study were deposited in the National Center for Biotechnology Information (NCBI) Gene Expression Omnibus (GEO) database with accession number GSE 225323. The data will be kept private until Aug 31, 2023, and the reviewer token is cvangueqnjoblgt.

## Ethics statement

The studies involving human participants were reviewed and approved by Ethics Committee of Tongji Hospital (No. TJ-IRB20210838). The patients/participants provided their written informed consent to participate in this study. The animal study was reviewed and approved by Tongji Hospital Guide for the Care and Use of Laboratory Animals (approval number: TJ-202111004).

## Author contributions

YL conducted the experiment and drew the manuscript. LC and NG conducted the animal experiment. CL isolated extracellular vesicles from the oviductal fluid. MW helped with bioinformatical analysis. LZ and FL helped to collect oviductal fluid samples. CS and LJ designed the experiment. All authors contributed to the article and approved the submitted version.
