# Supplementary material for: Oviductal extracellular vesicles from women with endometriosis impair embryo development
Source: Front Endocrinol (Lausanne). 2023 Jun 20;14:1171778. doi: 10.3389/fendo.2023.1171778 (PMC10319124; doi:10.3389/fendo.2023.1171778)
Supplement: Supplementary file 2 [file Table_1.docx]

Supplementary Table 1 Primer Sequences

| *Uqcrh-F* | AGGACGAACGAAAGATGC |
| --- | --- |
| *Uqcrh-R* | CGGGAAGACACGCGATTA |
| *Ndufa4-F* | AAGCCAAGAAGCATCCCA |
| *Ndufa4-R* | TGCCAAGCGCATCACATA |
| *Cox7c-F* | CGTCGCAGCCACTATGAG |
| *Cox7c-R* | AACCGCCACTTGTTTTCC |
| *Atp5l-F* | GTTCATCCGTAACTTCGC |
| *Atp5l-R* | CACCAAACCATTCAGCAC |
| *Trp53-F* | TGAACCGCCGACCTATCC |
| *Trp53-R* | GGCAGGCACAAACACGAA |
| *Fasl-F* | CACCGCCATCACAACCAC |
| *Fasl-R* | CCAGAGCCACCAGAACCA |
| *Gapdh-F* | TGCACCACCAACTGCTTAG |
| *Gapdh-R* | GATGCAGGGATGATGTTC |

Supplementary Table 2. Clinical Characteristics of recruited patients

| Sample | Age, years | Experimental group | BMI | Gravidity | Parity | Surgery date from LMP / length of the cycle | Estimated menstrual cycle phase | CIN grade | EMT |
| --- | --- | --- | --- | --- | --- | --- | --- | --- | --- |
| 1 | 39 | Control | 19.38 | 2 | 1 | 22/30 | Secretory | Ⅲ | - |
| 2 | 41 | Control | 22.52 | 1 | 1 | 19/31 | Secretory | Ⅲ | - |
| 3 | 39 | Control | 21.78 | 2 | 2 | 16/28 | Secretory | Ⅲ | - |
| 4 | 36 | Control | 21.51 | 4 | 3 | 19/35 | Secretory | Ⅲ | - |
| 5 | 43 | Control | 21.08 | 4 | 2 | 15/29 | Secretory | Ⅲ | - |
| 6 | 34 | Control | 23.31 | 3 | 1 | 18/32 | Secretory | Ⅲ | - |
| 7 | 41 | Control | 21.09 | 4 | 1 | 17/31 | Secretory | Ⅲ | - |
| 8 | 34 | Control | 22.86 | 2 | 2 | 19/31 | Secretory | Ⅲ | - |
| 9 | 39 | Control | 23.83 | 2 | 1 | 14/30 | Secretory | Ⅲ | - |
| 10 | 41 | Control | 21.72 | 1 | 1 | 8/28 | Proliferative | Ⅲ | - |
| 11 | 38 | Control | 19.92 | 2 | 1 | 12/32 | Secretory | Ⅲ | - |
| 12 | 35 | Control | 24.56 | 2 | 1 | 15/31 | Secretory | Ⅲ | - |
| 13 | 34 | Control | 23.63 | 3 | 1 | 21/28 | Secretory | Ⅲ | - |
| 14 | 34 | EMT | 23.63 | 3 | 1 | 19/37 | Secretory | - | Pelvic endometriosis with adenomyosis |
| 15 | 42 | EMT | 18.03 | 1 | 1 | 18/31 | Secretory | - | Pelvic endometriosis with adenomyosis |
| 16 | 37 | EMT | 19.20 | 1 | 1 | 29/35 | Secretory | - | Deep endometriosis |
| 17 | 40 | EMT | 25.00 | 2 | 1 | 13/28 | Secretory | - | Deep endometriosis |
| 18 | 42 | EMT | 22.66 | 3 | 1 | 9/30 | Proliferative | - | Pelvic endometriosis with adenomyosis |
| 19 | 36 | EMT | 23.04 | 1 | 1 | 17/30 | Secretory | - | Pelvic endometriosis with adenomyosis |
| 20 | 34 | EMT | 22.26 | 2 | 1 | 24/33 | Secretory | - | Pelvic endometriosis with adenomyosis |
| 21 | 42 | EMT | 21.56 | 3 | 1 | 12/28 | Proliferative | - | Pelvic endometriosis with adenomyosis |
| 22 | 37 | EMT | 26.03 | 1 | 1 | 18/34 | Secretory | - | Deep endometriosis |
| 23 | 36 | EMT | 24.6 | 1 | 1 | 16/30 | Secretory | - | Pelvic endometriosis with adenomyosis |
| 24 | 39 | EMT | 28.4 | 3 | 2 | 17/30 | Secretory | - | Pelvic endometriosis with adenomyosis |
| 25 | 41 | EMT | 20.39 | 4 | 1 | 19/34 | Secretory | - | Pelvic endometriosis with adenomyosis |
| 26 | 35 | EMT | 23.49 | 1 | 1 | 15/28 | Secretory | - | Pelvic endometriosis with adenomyosis |
| 27 | 36 | EMT | 23.72 | 2 | 1 | 22/27 | Secretory | - | Pelvic endometriosis with adenomyosis |

Supplementary Table 3. Clinical Characteristics of recruited patients, mean ± SEM

|  | Control (n = 13) | EMT (n=14) | P |
| --- | --- | --- | --- |
| Age (years) | 38 ± 0.8623 | 37.93 ± 0.8015 | 0.9520 |
| BMI | 22.09 ± 0.4267 | 23 ± 0.7215 | 0.2977 |
| Gravidity | 2.462 ± 0.2912 | 2 ± 0.2774 | 0.2618 |
| Parity | 1.385 ± 0.1804 | 1.143 ± 0.09705 | 0.2398 |

EMT, endometriosis; BMI, body mass index.
